# Drivers of Efficiency Breakthroughs: Key Technological Advances in Monolithic Perovskite/Silicon Tandem Solar Cells

**DOI:** 10.3390/nano16090540

**Published:** 2026-04-29

**Authors:** Yang Sun, Zijuan He, Yushuai Xu, Kun Chen, Haiwen Peng, Bin Chen, Ruicun Yue, Shizhong Yue, Haipeng Yin, Zi Ouyang

**Affiliations:** 1 JA Solar Technology Yangzhou Co., Ltd., Yangzhou 225131, China; hezj@jasolar.com (Z.H.); xuyushuai@jasolar.com (Y.X.); yz.chenkun@jasolar.com (K.C.); penghaiwen@jasolar.com (H.P.); chenbin@jasolar.com (B.C.); yinhp@jasolar.com (H.Y.); ouyangzi@jasolar.com (Z.O.); 2 BaoTou JA Solar Technology Co., Ltd., Baotou 014030, China; yuerc@jasolar.com; 3 Laboratory of Solid−State Optoelectronics Information Technology, Beijing Key Laboratory of Low Dimensional Semiconductor Materials and Devices, Institute of Semiconductors, Chinese Academy of Sciences, Beijing 100083, China; yueshizhong@semi.ac.cn

**Keywords:** perovskite–silicon tandem solar cells, power conversion efficiency, stability, interconnection layer, crystalline silicon bottom−cell, optical management

## Abstract

Crystalline silicon solar cells have long dominated the global photovoltaic market due to their mature manufacturing processes, excellent stability, and abundant raw material reserves, accounting for over 90% of the total PV market share. However, single−junction c−Si solar cells are approaching the Shockley–Queisser (SQ) efficiency limit of ~29.4%, creating an urgent need for next−generation PV technologies to achieve higher power conversion efficiency (PCE). Monolithic perovskite/silicon tandem solar cells (PSTSCs) stand as the most commercially promising technology to surpass the single−junction efficiency limit. Since their first demonstration in 2015, PSTSCs have experienced rapid technological advancement, with the certified PCE reaching 35.0% in 2026. This review posits that their rapid efficiency ascent is not serendipitous but driven by synergistic innovations across critical subsystems. We systematically deconstruct these efficiency drivers, encompassing top−cell materials, bottom−cell architecture, and optical management. We conclude by outlining future research frontiers essential for transforming this lab−champion technology into a mainstream energy solution.

## 1. Introduction

The urgent need to combat climate change and achieve carbon neutrality has accelerated global research into high−efficiency, low−cost photovoltaics. Among the many emerging solar cell technologies (e.g., CdTe, CIGS, organic solar cells, nanostructured systems [1,2]), crystalline silicon solar cells have long dominated the photovoltaic market thanks to their mature industrial chain and continuous technological innovation. In 2025, the annual installed power capacity of crystalline silicon photovoltaic modules exceeded 500 GW [3]. The introduction of structures such as tunnel oxide passivated contact (TOPCon) and heterojunction (SHJ) has enabled crystalline silicon cells to continuously approach their theoretical limit (approximately 29.4%) [4]. Due to the relatively low manufacturing cost, TOPCon solar cells have gradually formed the largest production capacity of crystalline silicon modules. The core advantage of this technology lies in the selective transport effect of the ultra−thin tunnel oxide on the back surface and the doped poly−silicon thin film. For n−type TOPCon solar cells, when the n^+^ doped thin silicon layer (usually a composite silicon layer or a poly−silicon layer) is in contact with the n−type substrate, it causes the energy band of the substrate to bend downward, reducing the electron transport barrier. The presence of the ultra−thin oxide layer allows only majority carriers (electrons) to pass through the passivation structure and be collected by the back surface electrode, while blocking minority carriers (holes), thereby separating holes and electrons and further reducing back surface recombination. Currently, the efficiency of n−type TOPCon cells has reached 26.55% [5].

Another mass production technology is the SHJ solar cell. The structure of SHJ solar cells includes carefully arranged intrinsic and in situ doped a−Si: H thin films on the surface of crystalline silicon, with transparent conductive oxide (TCO) coatings and screen−printed metal electrodes on both sides. The intrinsic (i) a−Si: H layer is located between the c−Si wafer and the doped a−Si: H layer. It serves as the passivation layer for the front surface emitter and the back surface, while minimizing the loss of Voc. The n−type and p−type doped a−Si: H thin films promote carrier selectivity through the energy band bending and inversion regions at their respective contact interfaces, thereby facilitating lateral charge transfer with the TCO layer. The doping level of these thin films is crucial for optimizing the built−in electric field and maximizing device efficiency. Currently, the efficiency of n−type SHJ cells has reached 26.8%, with an open−circuit voltage exceeding 751 mV. However, due to the fixed bandgap of crystalline silicon materials (approximately 1.12 eV), the efficiency improvement of single−junction cells has entered a plateau, and new device architectures are urgently needed to break through the SQ limit.

Meanwhile, metal halide perovskite solar cells, with their excellent photoelectric properties (such as high light absorption coefficient, and long carrier diffusion length) and the advantage of low−cost solution processing, have achieved a leap in efficiency from 3.8% to over 27.3% in just over a decade [5]. The main reason for the efficiency increase to >27% is the synergistic optimization of the interface [6]. In terms of hole selectivity, compact and chemically stable self−assembled monolayers (SAMs) suppress interface recombination by ensuring complete molecular coverage and minimizing energy level offsets. Dipole−engineered carbazole SAMs reduce the valence band offset to below 0.1 eV while stabilizing the favorable orientation of perovskite at the buried interface. In terms of electron selectivity, the ion−shielding intermediate layer transforms C_60_ from a passive electron transport layer into an active barrier that prevents ion redistribution. More importantly, the bandgap of perovskite materials can be continuously adjusted (approximately 1.2–2.3 eV), enabling them to form an ideal tandem structure with crystalline silicon cells. The development history of perovskite/crystalline silicon (perovskite/Si) tandem solar cells stands as one of the most compelling scientific narratives in photovoltaic (PV) technology over the past decade. Its evolutionary trajectory vividly illustrates leapfrog breakthroughs, from fundamental concept validation to the gradual approximation of theoretical efficiency limits [7].

The core objective of tandem solar cells is to surpass the SQ efficiency limit of single−junction photovoltaic devices [8]. The fundamental principle involves stacking semiconductor materials with different bandgaps to achieve a broader and more efficient utilization of the solar spectrum. Although solar irradiance spans a wide wavelength range, from ultraviolet to near−infrared, a single semiconductor material can only efficiently absorb photons with energy exceeding its bandgap; photons with energy below the bandgap pass through unutilized (transmission loss), while those with energy far above the bandgap dissipate excess energy as heat (thermalization loss). The tandem architecture addresses this by positioning a wide−bandgap material as the top cell to absorb high−energy photons (e.g., visible light) while transmitting low−energy photons (e.g., near−infrared light). A narrow−bandgap material serves as the bottom cell to capture these transmitted long−wavelength photons. This spectral segmentation strategy mitigates the two primary energy loss mechanisms, theoretically boosting PV conversion efficiency to over 45% [9].

The pairing of perovskite and crystalline silicon is widely regarded as an ideal combination for high−efficiency tandem cells, owing to their exceptional complementarity across physical properties, process compatibility, and industrial foundations. First, in terms of spectral matching: crystalline silicon, a mature PV material, has a bandgap of ~1.12 eV and exhibits a strong response to wavelengths below 1100 nm (encompassing near−infrared and visible ranges). To achieve optimal current matching with silicon, the top cell requires an ideal bandgap of 1.68–1.75 eV. Organic–inorganic hybrid perovskite materials (e.g., derivatives of MAPbI_3_ or FAPbI_3_) offer tunable bandgaps via precise halogen component (iodine, bromine, chlorine) regulation [10], enabling tailoring to the target ~1.68 eV—perfectly matching the silicon bottom cell. Second, perovskite materials possess a suite of superior PV properties: they exhibit an ultra−high light absorption coefficient, such that a film merely hundreds of nanometers thick can nearly fully absorb above−bandgap solar photons [11]. Additionally, perovskites demonstrate long carrier diffusion lengths and low defect densities (following appropriate passivation), ensuring efficient separation and transport of photogenerated electrons and holes to electrodes, thereby enabling high open−circuit voltages (Voc) and fill factors (FFs). For instance, through n−type quinoxalinephosphine oxide−based cathode interfacial layers, the Voc of wide−bandgap perovskite single−junction cells has been elevated to 1.31 V [12], laying a robust foundation for high−efficiency tandem cells. Third, from the perspective of manufacturing processes and industrialization, the perovskite/Si tandem solution offers unique advantages. The crystalline silicon PV industry, with decades of development, has established a global, highly mature, and cost−declining industrial chain. By directly depositing perovskite top cells onto existing high−efficiency silicon cells (e.g., heterojunction (SHJ) or TOPCon cells), this infrastructure can be fully leveraged. Critically, high−quality perovskite films can be fabricated via low−temperature solution processes [13] (e.g., slot−die coating [14,15,16], inkjet printing [17]) or vapor deposition [18]. In practical manufacturing, the thermal processing temperature of perovskite modules is typically maintained below 150 °C. This aligns with the low−temperature processing requirements of heterojunction silicon cells, avoiding damage to the silicon cell’s passivation layer from high−temperature treatments. Such process compatibility significantly reduces the complexity and cost of technology integration.

**Figure 1:**
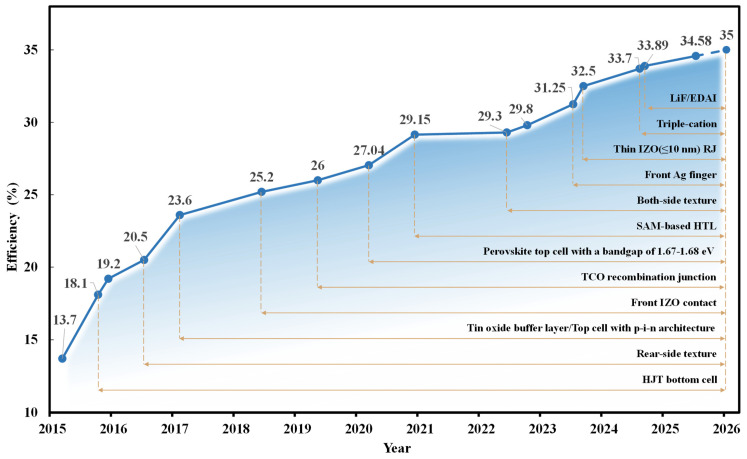
Literature−reported trends in the evolution of PSTSCs champion efficiency (area: ~1 cm^2^, stable efficiency). Dotted lines are used to indicate techniques that were consistently applied during the efficiency breakthrough.

The origin of perovskite/silicon tandem solar cells can be traced back to 2015, when researchers successfully constructed a monolithic perovskite/silicon tandem structure for the first time, initially verifying the feasibility of exceeding the theoretical limit (approximately 29.4%) of single−junction silicon cells [19]. In the early stage, the efficiency of devices was generally below 20%, mainly limited by the performance bottleneck of the wide−bandgap perovskite top cell. In particular, its Voc was far lower than the theoretical value, which was due to severe non−radiative recombination losses and unfavorable interfacial energy band alignment. Meanwhile, it was difficult to strike a balance between electrical conductivity and optical transparency in the recombination layer connecting the top and bottom cells, which became a key factor restricting the FF and short−circuit current (Jsc). Through material composition regulation and interface modification, the device structure was gradually optimized. In 2016, the laboratory efficiency of perovskite/silicon tandem cells exceeded 20% for the first time, reaching 20.5% [20]. However, problems such as poor device stability and large efficiency fluctuations still remained prominent, and a systematic technical system had not yet been established. As shown in Figure 1, since 2017, the efficiency of perovskite/crystalline silicon tandem cells has started to increase rapidly and steadily, and the breakthroughs in core technologies have shown diversified characteristics. At the material level, there have been innovations in the wide−bandgap perovskite material system [21], and the defect passivation strategies [22] have become more mature. At the interface level, technologies such as atomic−scale interface bonding [23] and multifunctional interface modification have been widely applied, effectively suppressing non−radiative recombination. At the structure level, p−i−n architecture [21], antireflection structures, and ultra−thin TCO layers [24] have enabled the effective utilization of sunlight. In this process, material systems, device structures, and preparation methods with excellent performance have been gradually accumulated and widely applied, driving the efficiency of perovskite/crystalline silicon tandem cells to exceed 33% in 2024 [25].

In this review, we focus on solutions for improving efficiency, especially those achieving over 33% (Table 1). We systematically review the key factors that have driven significant leaps in the conversion efficiency of perovskite/crystalline silicon tandem solar cells in the short term. Beyond simply listing high−efficiency reports, we critically analyze the underlying material design strategies—including perovskite composition engineering, defect passivation, the optimization of hole transport layers, the tailoring of silicon bottom cells, and optical management—as well as the device physics principles that must be followed to realize high−efficiency tandem solar cells. Finally, we summarize the remaining issues that constrain the performance of perovskite/silicon tandem solar cells, such as interface recombination, light−induced degradation, halide segregation, and scaling−up challenges, and attempt to identify directions for further enhancing performance stability and promoting industrial applications. This review distills the common principles behind record−breaking devices and offers clear guidelines on material selection and device architecture to accelerate progress toward 35% and beyond.

## 2. Driver 1: The Evolution of Wide−Bandgap Perovskite Top Cells

As the light−absorbing front of monolithic perovskite/silicon tandem solar cells, the performance of perovskite top cells directly determines the Voc and Jsc of the entire device. In recent years, this sub−cell has achieved a series of breakthroughs in material composition design, film forming process, and defect passivation, which together constitute the core driving force for the leap in efficiency.

### 2.1. The Bandgap–Voltage Trade−Off: The Fundamental Challenge

In the monolithic perovskite/crystalline silicon tandem solar cells, selecting the bandgap of the perovskite top cell around 1.68 eV is a balance point derived from a combination of theoretical calculations and experimental optimization. This value is not arbitrarily determined but is chosen to maximize the efficiency potential of the tandem cells while comprehensively considering various limitations in actual fabrication.

The core reasons can be summarized as follows: First, achieving perfect current matching. This is the most fundamental reason. The top and bottom sub−cells of the monolithic tandem cells are connected in series, and the output current of the entire cell is limited by the smaller of the two. Therefore, the currents generated by the top cell and the bottom cell must be made almost equal to maximize the total output. The bandgap of the crystalline silicon bottom cell is fixed (approximately 1.12 eV). To achieve current matching with it, the theoretically optimal bandgap of the top cell is in the range of 1.7–1.8 eV [42]. More refined simulation calculations have found that considering the actual illumination conditions and the luminescent coupling effect inside the cell (i.e., the light emitted by the top cell can be reabsorbed and utilized by the bottom cell), this optimal bandgap will shift slightly down to the range of 1.64–1.72 eV, and 1.68 eV is right at the center of this optimal range [43]. Second, balancing voltage, current, and stability. Although a slightly higher bandgap (e.g., above 1.72 eV) may make it easier to achieve current matching, it introduces new problems in actual materials, making 1.68 eV a better choice: Wide−bandgap perovskites generally have a large Voc loss, meaning that the actual voltage obtained is far lower than the theoretical value. The higher the bandgap, the more severe this problem usually is. Selecting 1.68 eV is a compromise and optimization of the voltage loss while pursuing high current, in order to obtain the best actual output power. In addition, to achieve a wide bandgap, bromine (Br) and iodine (I) need to be mixed into the perovskite simultaneously. However, under illumination or an electric field, halogen ions will migrate, which seriously damages the stability. Therefore, 1.67–1.68 eV is a critical window, and under this bandgap, strategies such as interface engineering can more effectively suppress this harmful phase separation.

To move beyond qualitative descriptions, we here provide a quantitative framework for evaluating the key loss mechanisms in wide−bandgap perovskite top cells. For a perovskite with a bandgap of Eg ≈ 1.68 eV, the radiative limit of the open−circuit voltage (V_OC,rad_) at 300 K under 1 sun illumination is approximately 1.38 V, as calculated from the detailed balance model. The actual V_OC_ achieved in state−of−the−art devices is around 1.3 V, corresponding to a total voltage deficit ΔV_total_ = V_OC,rad_ − V_OC_ ≈ 80 mV. This deficit can be further decomposed into two components: (i) non−radiative recombination loss ΔV_non−radiative_ = (k_B_T/q) ln(1/PLQY), where PLQY is the photoluminescence quantum yield; and (ii) radiative loss due to incomplete absorption and escape of photons, which is typically small for high−quality films. For a device with PLQY = 5% (a typical value for optimized wide−bandgap perovskites), ΔV_non−radiative_ = 77 mV. The remaining ~3 mV arises from interfacial recombination and transport losses, particularly at the perovskite/C_60_ interface. Using quasi−Fermi level splitting (QFLS) measured by absolute photoluminescence, one can directly quantify the upper limit of V_OC_ before charge extraction. In record perovskite/silicon tandems, QFLS values exceeding 1.34 eV have been reported, indicating that the actual V_OC_ is limited not by bulk recombination but by selective contact losses. Quantitatively, reducing the interfacial recombination velocity from 10^4^ cm/s to 10^3^ cm/s can increase V_OC_ by approximately 50 mV, as confirmed by drift–diffusion simulations. These numbers highlight that the primary bottleneck for further efficiency gains is no longer the bulk perovskite quality but rather the passivation of the buried interface and the optimization of the SAM/perovskite energy level alignment. For current matching, the sensitivity of tandem efficiency to a current mismatch ΔJ = |J_sc, top_ − J_sc, bottom_| can be expressed as ΔPCE/PCE ≈ −(ΔJ/J_mp_), where J_mp_ is the current at the maximum power point. For a tandem with J_mp_ ≈ 20 mA/cm^2^, a mismatch of 0.5 mA/cm^2^ reduces the efficiency by about 2.5% relative (≈0.8 absolute percentage point), underscoring the need for precise bandgap tuning and optical management. Such quantitative benchmarks provide actionable targets for future material and device engineering.

### 2.2. Compositional Engineering for Performance and Stability

Precise regulation of wide−bandgap (WBG) perovskite materials is a prerequisite for achieving efficient current matching. Limited by the maturity of the material system, methylammonium lead iodide, with a bandgap of around 1.61 eV, was used in early papers, resulting in a low open−circuit voltage of less than 1.72 V [19,20,44]. Research in this field has evolved from simple binary or ternary mixtures in the early stage to complex multi−component collaborative design today. For instance, the introduction of triple−cation systems (Cs/MA/FA) by Saliba et al. significantly suppressed phase impurities and enabled highly reproducible high−efficiency devices in single−junction configurations [45]. Subsequently, the incorporation of additional A−site elements such as Rb and K further optimized compositional stability [38,46]. The introduction of a third halide (Cl) into the X−site to form triple−halide systems suppresses Br/I phase segregation [47]. As mentioned earlier, the ideal bandgap of the top cell needs to be stably within the range of 1.68–1.74 eV to form the optimal spectral splitting with the crystalline silicon bottom cell. Compositional engineering, including cation engineering and anion engineering, is the core strategy for achieving this regulation.

Cation engineering mainly focuses on the regulation of A−site cations. Traditional formamidinium (FA^+^)−based perovskites are regarded as the foundation due to their near−ideal bandgap and excellent thermal stability. However, pure FAPbI_3_ tends to form a non−perovskite yellow phase (δ−phase) at room temperature, lacking photovoltaic activity. To stabilize its black phase (α−phase), researchers have introduced various auxiliary cations. Although the introduction of methylammonium (MA^+^) can effectively stabilize the structure, it brings thermal instability. The reason is that during the preparation of MA−based devices, tiny dust particles in the perovskite material can form pinholes in the indium tin oxide (ITO) layer, thus creating a channel for the escape of MA^+^ and ultimately leading to a decrease in efficiency [21]. Therefore, smaller inorganic cations such as cesium (Cs^+^) and rubidium (Rb^+^) play a key role. The incorporation of Cs^+^ can not only effectively inhibit the formation of the yellow phase but also improve the crystallization quality of the thin film; while the addition of Rb^+^ has been proven to further passivate grain boundary defects and reduce non−radiative recombination centers [46]. The latest progress is reflected in the extreme optimization of the ratios of these cations and the exploration of new organic cations. Following Mariotti et al. and finely regulating the ratios of cations such as FA and Cs, a trihalide perovskite with a bandgap of 1.68 eV was successfully prepared, and an open−circuit voltage of up to 1.28 V was achieved in combination with interface modification, which directly promoted the leap in the efficiency of tandem devices [48].

Anion engineering mainly achieves the widening of the bandgap by adjusting the ratios of X−site halogen anions (I^−^, Br^−^, Cl^−^). Iodine (I) provides a narrow bandgap, and the introduction of bromine (Br) is the most direct and effective method to widen the bandgap. However, a high proportion of Br can induce a serious phase segregation problem, that is, under the action of light or an electric field, the I−rich and Br−rich regions will spontaneously separate, resulting in dynamic changes in the bandgap and performance degradation [49]. This is one of the core challenges faced by wide−bandgap perovskites. The amount of chlorine (Cl) introduced is usually extremely small, but its role cannot be ignored [47]. It helps to improve the crystal growth kinetics and obtain thin films with larger grains and fewer defects. Xu et al. enhanced the solubility of chlorine by replacing some of the iodine with bromine to shrink the lattice parameter and reduced the lattice parameters by increasing the bromine content, thereby enhancing the solubility of chlorine, which further decreased the lattice parameters. A large amount of Cl is incorporated into the lattice, leading to a uniform halide distribution throughout the material with a reduction in lattice parameter and an increase in bandgap. As a result, the bandgap increased to 1.67 eV, and phase separation was also suppressed [50]. Mariotti et al. further optimized the trihalide perovskite and, in combination with PI interface, improved the band alignment, reduced non−radiative recombination losses, and enhanced charge extraction at the electron−selective contact. They achieved open−circuit voltages of up to 2.00 V and certified power conversion efficiencies of up to 32.5% in perovskite–silicon tandem solar cells [48].

To improve the stability of wide−bandgap perovskite, another research frontier lies in how to effectively suppress phase segregation while achieving a high Br content to meet the bandgap requirements. One strategy is through the synergistic effect of cation engineering and anion engineering, using the lattice strain generated by specific cations (such as large−volume organic cations) to pin halogen ions and prevent their migration [9]. Another strategy is to develop new halide precursors or additives to regulate the crystallization process from the precursor solution and form a more uniform and stable halogen distribution. Han et al. achieved a uniform phase distribution of wide−bandgap perovskites by introducing a melamine iodide (MLAI) additive: MLAI reduced the formation energy of the target perovskite phase (from −3.21 eV/unit to −3.76 eV/unit), making the target phase easier to form and suppressing the precipitation of heterogeneous phases. Kinetic regulation: reducing the nucleation barrier (from 0.42 eV to 0.12 eV) and delaying the crystallization process to provide a window for the formation of a uniform phase. Based on the optimized top cell, the stable efficiency of the tandem device reached 33.5%, the open−circuit voltage was 2.02 V, and the T_90_ lifetime exceeded 1100 h [36].

Although significant progress has been made, the phase segregation problem has not been fundamentally solved, especially under high light intensity and long−term working conditions. This remains a key bottleneck restricting the long−term stability of devices, and more fundamental material design strategies or passivation mechanisms need to be developed. Secondly, the compatibility problem between component uniformity and large−area preparation is prominent. The small−area thin films prepared by the laboratory spin−coating method have uniform components, but when scaled up to the module level, the drying kinetics of the solution method will lead to component gradients, affecting the performance consistency.

### 2.3. Preparation Techniques for Perovskite Film

**Figure 2:**
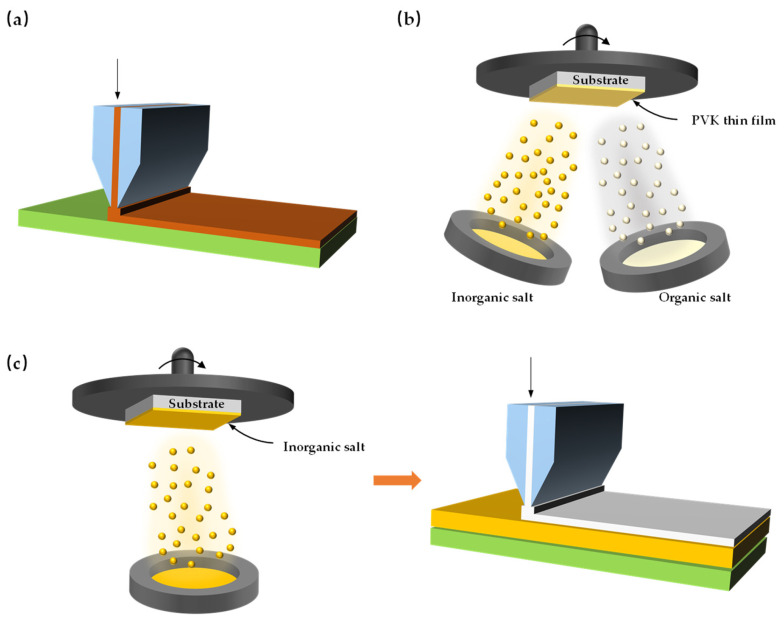
(**a**) Fully solution process, (**b**) fully vaporation process, (**c**) evaporation–solution hybrid process.

The preparation technique for perovskite films is a critical factor in achieving high−efficiency perovskite/crystalline silicon tandem solar cells. Different preparation methods directly influence film morphology, crystallinity, coverage, and ultimately device performance. As shown in Figure 2, perovskite film preparation techniques can be classified into three main categories: solution−based methods, vapor−based methods [51] (such as co−evaporation, sequential evaporation), and hybrid solution–vapor methods [52]. Solution−based methods include spin−coating, blade−coating, slot−die coating, and inkjet printing. Among these, spin−coating is the most widely used technique in laboratory research, though it suffers from low material utilization and poor scalability; blade−coating and slot−die coating are more suitable for large−scale production. Vapor−based methods such as thermal evaporation and chemical vapor deposition enable precise thickness control and high uniformity, making them particularly attractive for conformal coverage on micron−textured silicon substrates. Hybrid methods, such as vapor−assisted solution processing, combine the simplicity of solution−based approaches with the controllability of vapor−based techniques. This section systematically reviews these preparation techniques and their application progress in tandem devices, with a focus on how each technique affects film quality, interfacial properties, and device efficiency.

**Figure 3:**
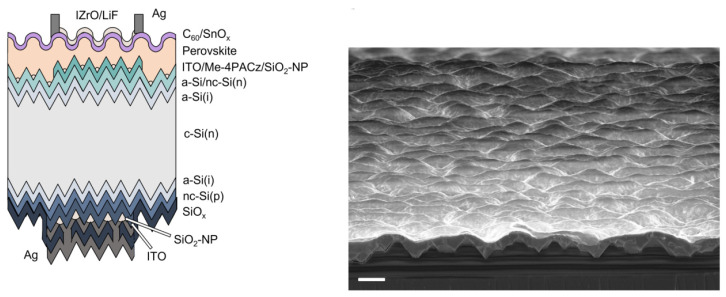
Cross−section schematic and tilted (10°) cross−section and top−view scanning electron microscope (SEM) images of the solution−processed perovskites on fully textured SHJ bottom cells [26]. The scale bars are 2 μm in width.

Among them, the fully solution process is widely adopted due to its low cost and simple operation, and is particularly suitable for the rapid verification of small−area laboratory devices. Combined with the reverse solvent engineering [53,54] or vacuum−flash evaporation [55,56,57,58], it can effectively control the crystal nucleation and growth kinetics, thereby obtaining a dense, large−grain, and low−defect high−quality film. The fully solution process has the highest efficiency of 34.58% [28]. Regarding the problems of the solution method and how it is difficult to grow on the full−textured silicon substrate, there have been some advancements. Turkay et al. used SiO_2_−NP to increase the wettability of the silicon substrate, enabling the perovskite film to form a conformal film on the silicon pyramids up to 2 μm in size (Figure 3). Using this structure, the perovskite–silicon tandem device achieved a PCE of 33.28% [26]. By using the solution method through the collaborative engineering of perovskite components (high−cesium−content wide−bandgap perovskite) and nc−Si: H TRJ, Yu et al. successfully fabricated a highly conformal and fully covered perovskite film on the full−texture SHJ bottom cell. This tandem cell achieved a remarkable PCE of 33.38% (certified efficiency 32.94%) and set a record of 21.21 mA/cm^2^ short−circuit current density [37]. The advantages of the solution method, such as low cost, simple process, and strong compatibility, make it the mainstream route for current laboratory and industrial exploration, promoting continuous improvement in tandem solar cell efficiency [59].

The vapor deposition film formation process uses the vapor of perovskite raw materials as the precursor and deposits the vapor onto the surface of the silicon substrate through physical or chemical methods, and then undergoes condensation and crystallization to form a perovskite film. This process has the advantages of good film uniformity, strong controllability of thickness, and no solvent residue, which can effectively solve the interface defects caused by solvent residue, and is suitable for the preparation of high−precision tandem solar cells. Currently, the vapor deposition film formation processes suitable for tandem solar cells include the thermal evaporation method and chemical vapor deposition (CVD) method [60]. Hou et al. used the dual−functional molecule TFPTMS to balance the deposition rates of organic and inorganic components, and based on the evaporation method, achieved a photoelectric conversion efficiency of 31.3% [18]. However, this process has problems such as high equipment cost, low preparation efficiency, and low raw material utilization rate; thus it is mainly used for the preparation of high−precision devices in the laboratory.

The evaporation–solution hybrid process combining vapor deposition and solution treatment provides an attractive approach for large−scale and high−performance perovskite absorption layers, especially in the demanding structure design of PSTSCs. Werner et al. were the first to apply the evaporation–solution hybrid process to PSTSCs [44]. By taking advantage of uniform vapor scaffolds and flexible solution chemistry, they overcome the key challenges related to textured substrates and large−area uniformity. Luo et al. utilized the synergistic effect of MCl and MASCN in the crystallization process to achieve a more uniform deposition of perovskite films with larger grain sizes and higher crystallinity, while reducing non−radiative recombination and inhibiting trap density and phase separation. By using an industrial double−sided textured SHJ with pyramids exceeding 3 µm, the efficiency reached an impressive 28.9% (active area of 1.05 cm^2^), and it was also demonstrated that this strategy is compatible with the scaling process using the blade coating method, achieving a 25.1% efficiency (aperture area of 16 cm^2^) [61]. However, there are still some issues: (1) The diffusion and reaction kinetics between the precursor vapor and the solution salt must be carefully controlled to avoid unreacted scaffolds or residual components. (2) To expand to large substrates, uniform liquid surface control, blade speed optimization, and solvent drying management are still required. With the continuous increase of high−efficiency reports and the in−depth understanding of process dynamics, the hybrid deposition method is expected to accelerate the transition of PSTSC from the laboratory to the factory [13].

### 2.4. Defect Passivation: From Bulk to Surface

**Figure 4:**
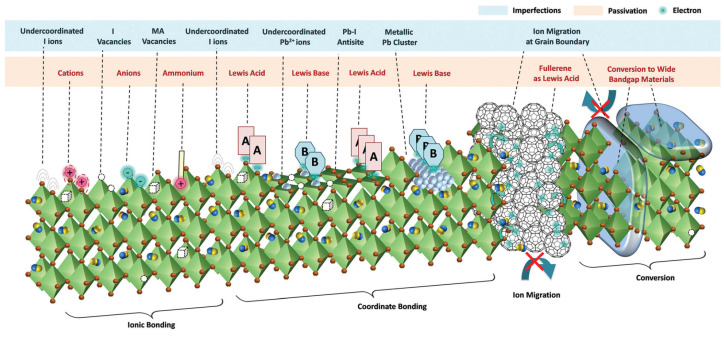
Defect types and passivation methods of perovskite top cell [62].

The performance bottleneck of perovskite/crystalline silicon tandem solar cells mainly stems from non−radiative recombination losses. Passivation technologies, including bulk passivation and interface passivation (Figure 4), are the core means to suppress these losses and enhance the Voc and FF of the devices.

Bulk passivation aims to eliminate or passivate point defects within the perovskite thin film and at the grain boundaries, such as uncoordinated lead ions (Pb^2+^), halogen vacancies (V_x_), and interstitial ions [63]. These defects act as Shockley–Read–Hall (SRH) recombination centers, significantly reducing the carrier lifetime. The current mainstream bulk passivation strategies rely on additive engineering. Small−molecule passivators (such as Lewis base compounds) coordinate with uncoordinated Pb^2+^ through their lone pair electrons, effectively filling deep−level trap states. The latest progress is reflected in the design of multifunctional passivators, such as molecules with the capabilities of passivation, hydrophobicity, and lattice strain regulation [64,65]. Chen et al. added two additives, MACl and MAH_2_PO_2_, to the perovskite precursor, which can significantly improve the grain morphology of wide−bandgap (1.64–1.70 electron volts) perovskite thin films, thereby increasing the photocurrent of the solar cells and reducing the Voc loss. The addition of MACl increases the grain size, while MAH_2_PO_2_ reduces non−radiative recombination by passivating the perovskite grain boundaries, and the two functions work well in synergy [66]. Yang et al. used 2−fluoroisonicotinic acid (2−FNA), which has fluorine (−F), carboxylic acid (−COOH), and pyridine nitrogen as functional groups in the perovskite precursor. The trifunctional Lewis base additive passivates the perovskite layer to assist the crystallization process [67]. Yang et al., using halogen circulation agents (HCAs) of N−halosuccinimide molecules as the sustainable stabilizers, in order to achieve dynamic halogen equilibrium within the precursor solution and perovskite film, which blocks the migration path of Br^−^/I^−^ ions both in crystallization and aging of WBG perovskites [27]. Notably, in wide−bandgap triple−halide perovskites, the high bromine content exacerbates the formation of halogen vacancies. Therefore, the targeted introduction of cations (such as guanidinium ions [68]) that can strongly interact with halogen ions has become a new trend. In addition, Yang et al. found that the preferential nucleation of the bromine−rich phase during the thin−film formation process is the root cause of the instability of wide−bandgap perovskites. During the nucleation stage, the bromine−rich phase forms preferentially due to its thermodynamic advantage, causing compositional inhomogeneity, which in turn accelerates halide migration and phase separation under operating stress. Therefore, they introduced potassium thiocyanate (KSCN) as an additive to achieve dual regulation of thermodynamic inhibition and kinetic blocking. K^+^ increases the nucleation energy barrier of the bromine−rich phase, suppressing its preferential nucleation and achieving uniform co−nucleation of iodine and bromine. SCN^−^ coordinates with excess Pb at the grain boundaries to form a 2D perovskite phase (APb(SCN)_x_), increasing the ion migration energy barrier, blocking the ion migration channels at the grain boundaries, and passivating deep−level defects. The perovskite/silicon tandem solar cell achieves an efficiency of 33.08% (certified efficiency of 32.52%), with stable performance after 540 h of outdoor operation, and an extrapolated T90 lifetime of over 9700 h under continuous maximum power point tracking [38].

In the internal interfaces of tandem cells, the perovskite/electron transport layer (C60) interface is particularly important [69]. There is a high energy band offset between this type of material and the perovskite interface, which leads to difficulties in electron extraction and enhanced recombination. Moreover, the poor thermal stability of organic materials can easily trigger interface degradation. Adoptable interface passivation strategies, such as contact displacement, chemical passivation, surface−potential homogenization, and field−effect passivation, have promoted the rapid improvement of the efficiency of tandem cells [70]. Among them, the commonly used LiF mainly serves as a shunt barrier layer, whose function is similar to that of the thin and insulating silicon oxide layer previously used in thin−film silicon solar cells, which is used to block shunt paths and improve the FF [21]. Er−raji et al. employed PDAI to induce a positive dipole moment at the perovskite/C60 interface through work function engineering, reducing the conduction band offset (ΔE_C,ETL_) from 180 meV in the reference device to 70 meV in the target device. The key breakthrough lies in deep field−effect passivation, which extends electron accumulation to the entire bulk material of the perovskite absorption layer, increasing the average electron concentration in the absorption layer from the simulated 1 × 10^14^ to 4 × 10^15^ cm^−3^. This significantly enhances the conductivity of the absorption layer and reduces carrier transport losses, becoming the main driving force for the improvement of the FF. The device achieved a maximum PCE of 33.1% and an excellent Voc of 2.01 V, with a certified steady−state PCE of 31.6% [34]. Li et al. proposed a strategy involving an SbCl_3_−doped n−type 2D perovskite intermediate layer. By substituting the Pb^2+^ sites in the PEAI−based 2D perovskite with Sb^3+^ to achieve n−type doping, a dual−functional intermediate layer with both defect passivation and interface electric field regulation was constructed. This solution solves the problems of blocked carrier transport and energy band mismatch caused by quantum confinement in traditional 2D perovskite passivation layers. The efficiency of the fabricated perovskite/silicon tandem cell is 33.10% (certified 32.56%) [32].

### 2.5. Hole Transport Layers Tailored for Tandem Solar Cells

In recent years, self−assembled monolayers (SAMs) have driven performance breakthroughs by accelerating hole extraction, compared to traditional hole transport layers [71,72,73,74,75,76]. SAMs consist of three distinct parts: anchoring group, spacer group, and terminal group. The classification of SAMs is primarily based on two structural motifs: the anchoring group and the functional head group. Anchoring groups, including phosphonic acids (most commonly used, e.g., Me−4PACz, MeO−2PACz), carboxylic acids (e.g., TPA−PA, MC−43), and cyanoacetic acids (e.g., Cz−CA, MPA−Ph−CA), can form ultra−thin and uniform HTLs on the substrate (typically ITO or FTO) through chemical bond and passivate defects at the interface. The terminal groups of SAMs typically consist of functional head groups, including carbazole−core (Cz, most widely used), triphenylamine−core (TPA), phthalocyanine−core, and aza−helicene−core (e.g., A5HPA, A7HPA) [30], which can significantly enhance the performance and stability of devices by modulating their interfacial energy levels, passivating interfacial defects, improving crystalline quality, and thus enhancing interfacial stability. Their ultra−thin nature minimizes transmission losses while providing uniform coverage and chemical passivation to reduce interface non−radiative recombination. Record−performing perovskite/silicon tandem solar cells have utilized phosphonic acid−based SAMs to enhance hole selectivity. Wu et al. made a breakthrough in the design of SAM molecules by employing the D−A coplanar conjugation strategy to design open−shell molecules RS−1/RS−2, achieving stable intramolecular radical ion pairs and high spin concentration. The developed steric hindrance engineering inhibited the molecular stacking of the aromatic rings, enhancing the solution processability and assembly uniformity. The certified efficiency of the perovskite–silicon tandem cells based on RS−2 was 34.2%; in terms of stability, the efficiency remained above 97% after continuous operation at 45 °C for 2000 h, and it maintained 92% after 960 h in an 85 °C/60% humid heat environment [29]. Jia et al. developed a new type of asymmetric SAM molecule, HTL201. They designed the anchoring group (phosphate group) and the spacer group on the benzene ring sidechain of the carbazole core instead of using traditional nitrogen−atom bonding. Its vertical configuration reduces steric hindrance, enhances the interaction with the TCO layer, and forms a denser monomolecular coverage. Meanwhile, larger grains of perovskite grow on HTL201 with a more ordered orientation, effectively passivating interfacial defects. Benefiting from the suppression of interfacial non−radiative recombination and the increase in quasi−Fermi level splitting (QFLS), the device has a Voc as high as 2.001 V, and the champion device achieves an efficiency of 34.60% (certified efficiency of 34.58%) [28].

**Figure 5:**
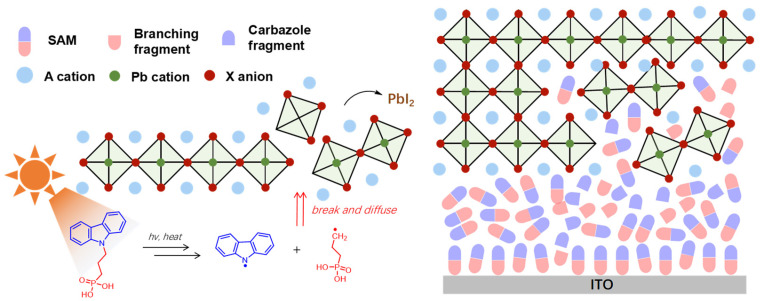
The influence of SAMs (3PACz) on the decomposition of perovskite during the aging process, and the proposed mechanism [30].

However, the challenges of SAM−based hole transport layers lie in the uneven coverage and disordered stacking of ultra−thin SAM layers on rough substrates, which hinder hole transport and exacerbate non−radiative recombination [77,78,79,80,81]. Additionally, SAMs have stability issues due to chemical decomposition, desorption, and weak interfacial adhesion (Figure 5). Yan et al. significantly improved the intrinsic stability of molecules against thermal aging, light, and electro−oxidation by designing A5HPA and A7HPA with non−planar π−conjugated systems. Among them, the A7HPA molecule significantly strengthens the intermolecular π−π interaction, thus constructing a rigid hole transport layer [30]. Luo et al. designed and prepared an HTL based on in situ cross−linking through the Schiff base reaction. By introducing the 4PACz−DM molecule with an amino group to react with the aldehyde−based cross−linker BPDA, a stable covalent network structure is formed. This cross−linked structure not only enhances the thermal stability of the molecular layer but also improves the contact quality at the perovskite interface, enhancing the interface passivation effect and the uniformity of the perovskite film. Experimental results show that the tandem cells using this cross−linked molecular contact layer achieve a conversion efficiency of over 34% on an area of 1 square centimeter and a certified efficiency of 33.61%. After approximately 1200 h of maximum power point tracking under 65 °C and AM1.5G illumination, three independent devices can still maintain 96.2 ± 1.7% of their initial efficiency, significantly outperforming cells with traditional monomer molecular layer structures [35]. Zhang et al. used silicon dioxide (SiO_x_) nanospheres to form local sub−micron contacts at the bottom of silicon pyramids, effectively solving the problem that it is difficult to uniformly cover SAM and deposit high−quality perovskite on traditional industrial textured silicon surfaces. This design not only maintains the light−trapping advantage of the silicon substrate but also significantly reduces interfacial recombination losses and improves carrier transport. By optimizing the filling concentration of SiO_x_ nanospheres (5 mg mL^−1^) and the perovskite bandgap (1.67 eV), a current−matched tandem device is obtained, achieving a certified conversion efficiency of 33.15% [31].

In conclusion, the progress of wide−bandgap perovskite top cells centers on resolving the trade−off among bandgap (~1.68 eV), open−circuit voltage, and phase stability. Compositional engineering (multi−cation, triple−halide) and advanced passivation (bulk/grain−boundary/interface) have pushed efficiencies beyond 34%, while self−assembled monolayers (SAMs) have replaced conventional HTLs by enabling nearly lossless hole extraction and record V~oc~ >2.0 V. However, intrinsic halide segregation under operation, non−uniform SAM coverage on textured surfaces, and scalability of green−solvent processing remain unsolved bottlenecks.

## 3. Driver 2: High−Performance Silicon Bottom Cells Reimagined

**Figure 6:**
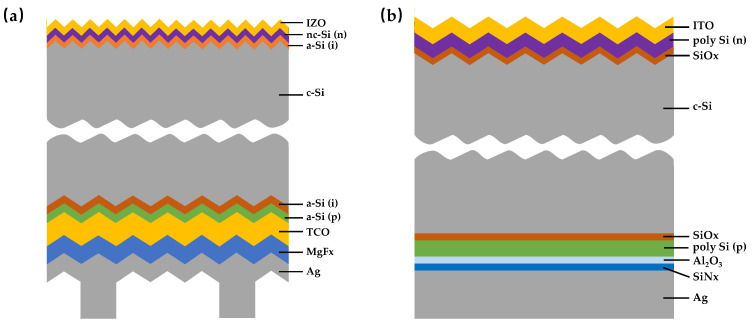
The configurations of different silicon bottom cells, (**a**) SHJ, (**b**) TOPCon.

The current development status of crystalline silicon bottom cells shows a clear trajectory of evolution from high−efficiency laboratory structures to industrially compatible platforms. At present, the mainstream technological routes for crystalline silicon bottom cells mainly include two types: SHJ and TOPCon (Figure 6) [82]. Among them, SHJ cells have become the preferred choice for constructing high−performance monolithic tandem cells due to their excellent surface passivation quality, high open−circuit voltage (Voc > 750 mV), and low−temperature process compatibility. The SHJ structure uses intrinsic and doped amorphous or nano−crystal silicon thin films to passivate the surface of crystalline silicon, effectively suppressing surface recombination and thus providing a foundation for high current and high voltage in tandem devices [83].

However, TOPCon technology is rapidly catching up through innovative strategies such as front surface texturing, double−sided passivated contact, advanced hydrogen passivation technology, and new interconnection layer design. Its long−term competitiveness in the field of tandem cells should not be underestimated. Nevertheless, the disadvantage of TOPCon cells lies in the insulating SiN_x_/AlO_x_ passivation layer on the front side. It is necessary to remove it first and then deposit TCO, which introduces additional optical absorption and contact resistance, weakening its original high−current advantage. This additional modification process also increases the complexity of the process and may cause damage to the silicon wafer surface. Therefore, it is very difficult to deposit a uniform perovskite thin film on a commercially textured TOPCon silicon substrate, and wet spots are likely to form, resulting in uneven film coverage. Currently, Wang et al. designed the asymmetric molecule 3−Me−4PACz as a new type of interconnection layer (ICL) to modify the surface of the metal oxide NiO_x_. By enhancing the wettability, uniformity, and adhesion of the ICL, wet spots were eliminated, and uniform growth of perovskite on the rough silicon surface was achieved. Utilizing the large dipole moment of this molecule, the interface energy offset was reduced, and the hole selectivity and charge extraction efficiency were improved. The perovskite/TOPCon tandem cell achieved a laboratory efficiency of 33.12% and a certified efficiency of 32.32%, setting a record. The open−circuit voltage was as high as 2.023 V, with a certified value of 2.015 V, the highest among all perovskite/silicon tandem cells [39].

The efficiency of perovskite/TOPCon tandem cells (~33%) is still lower than that of perovskite/SHJ tandem cells. Problems such as interface energy loss, low charge extraction efficiency, and poor interface adhesion limit their performance and stability. In terms of the latest progress, researchers are innovating and optimizing crystalline silicon bottom cells from multiple dimensions [84,85,86,87,88]. First, in terms of passivation quality, in response to the problem of weak field−effect passivation caused by insufficient boron doping in the p−type polycrystalline silicon (poly−Si) contact in the TOPCon structure, Ding et al. have developed a highly passivated TOPCon bottom cell and significantly improved its open−circuit voltage through processes such as AlO_x_−based hydrogenation, laying a foundation for realizing high−efficiency tandem cells [84]. Second, in terms of optical management, to overcome the difficulty of uniformly forming a perovskite thin film on the micron−scale pyramid−textured silicon surface, researchers have explored sub−micron or nanoscale surface texturing strategies. This fine texture can effectively trap light, enhance the infrared response, and provide a flatter growth substrate for perovskite, thus taking into account both optical gain and film quality [89,90]. Third, in terms of interconnection layer engineering, traditional ITO−based interconnection layers have problems such as sputtering damage and low−temperature process limitations. Therefore, a new type of polycrystalline silicon tunneling intermediate composite layer has been successfully developed. It not only avoids sputtering damage but also exhibits excellent electrical and optical properties, providing key support for high−efficiency and stable tandem devices [91].

In conclusion, silicon bottom cells have evolved from passive IR absorbers to active partners in tandem design. Although SHJ remains dominant due to superior passivation and low−temperature compatibility, TOPCon has recently achieved certified 33.12% efficiency via asymmetric SAM interlayers (3−Me−4PACz) that enable conformal perovskite growth on rough surfaces. Sub−micron texturing and poly−silicon tunneling recombination layers further decouple optical trapping from film quality. Nevertheless, the long−term operational stability of TOPCon−based tandems and the added processing complexity (removal of front SiN_x_/AlO_x_) require further validation.

## 4. Driver 3: Holistic Optical Management for Current Maximization

The core objective of light management strategies is to maximize the photon capture and utilization efficiency across the entire spectral range while minimizing optical losses. Efficient light management can not only enhance the Jsc but also indirectly improve the Voc and FF by optimizing the light field distribution, thereby driving the continuous increase in the overall power conversion efficiency. In recent years, technological advancements in this field have mainly manifested in three aspects: textured surface engineering, advanced antireflection coating design, and systematic optical optimization tailored to the characteristics of the tandem cells structure [92].

Textured surfaces serve as the cornerstone for enhancing light−capturing capabilities. For silicon−based solar cells, the adoption of pyramid−shaped or nanoscale textured surfaces can effectively reduce front surface reflection and extend the optical path through multiple light scatterings, significantly enhancing absorption in the near−infrared band. Harter et al. show that double−sided nanoscale textured silicon surfaces can not only increase the photocurrent of the bottom cell but also provide a more uniform incident light distribution for the perovskite top cell, thus improving current matching [93]. However, such rough surfaces pose significant challenges to the conformal deposition of perovskite thin films. To address this issue, Liu et al. have developed a two−step hybrid process of evaporation and solution, successfully achieving high−quality, pinhole−free perovskite thin film coverage on industrial textured silicon substrates, ensuring the integrity and uniformity of the top cell on complex three−dimensional structures [94]. This technological breakthrough makes it possible to integrate efficient light−capturing structures with high−performance top cells.

The design of antireflection coatings (ARCs) and transparent conductive oxide (TCO) window layers [95,96,97] is another crucial aspect. In monolithic tandem solar cells, the reflection of incident light needs to be more effectively suppressed because of the larger refractive index difference, due to a more detrimental air/IZO (n_air_:n_IZO_ = 1:~2) interface in the tandem compared to the air/glass (n_air_:n_glass_ = 1:1.5). Since the first tandem solar cell article, the LiF antireflection layer has been used [19]. The ARC located at the front of the device must achieve the lowest reflectance within a broad spectral range (typically 300–1200 nm). Albrecht et al. fabricated light management foils using LM foil and attached them to the light−incident side to reduce reflection losses [98,99]. Applying the LM foil leads to a significantly higher Jsc by more than 1 mA/cm^2^. By precisely controlling the thickness and refractive index of TCO materials such as Zn−doped In_2_O_3_ (IZO), an effective optical interference effect can be formed with subsequent functional layers to further suppress reflection losses. Kohnen et al. began to attempt to reduce the parasitic absorption of TCO. They verified that reducing the thickness of the top TCO electrode to 60 nm could result in J_pero + si_ > 40 mA/cm^2^ even under the condition of a polished front surface. However, thinning the IZO needs to be coordinated with the metal gridlines, and the resistivity and shading losses need to be balanced [100]. Wang et al. improved the crystallinity of IZO through low−temperature in situ annealing (optimal at 75 °C), reduced oxygen vacancies, and balanced conductivity, transparency, and mechanical stability. After annealing, the carrier mobility of IZO increased to 50.7 cm^2^·V^−1^·s^−1^, the sheet resistance decreased to 100 Ω/□, and the optical absorption decreased. Meanwhile, the increased crystallinity enhanced the mechanical toughness, reduced the halogen diffusion paths, and improved the device stability [40].

Therefore, systematic optical simulation and collaborative design are the keys to achieving global optimization. The light management of tandem solar cells is not simply the superposition of the optical designs of individual sub−cells but a complex process that requires global optimization. This includes precisely adjusting the bandgap of the top cell to achieve optimal spectral splitting, optimizing the thickness of the interconnection layer to balance its optical transparency and electrical performance, and minimizing the parasitic absorption of each functional layer (such as the hole transport layer and electron transport layer). For example, although the interconnection layer requires high conductivity, its material may absorb light in the visible region. Therefore, its thickness must be controlled within the optical dead zone, or the principle of destructive interference should be used to offset its absorption effect. Advanced optical models can accurately simulate the propagation, interference, and absorption behavior of light in multi−layer film systems, providing theoretical guidance for experimental design and ensuring that the entire solar spectrum from ultraviolet to near−infrared can be efficiently utilized by the two sub−cells.

Moreover, light management strategies for tandem photovoltaic devices must comprehensively account for real−world power generation conditions. Significant discrepancies exist between field operating environments and standardized laboratory test conditions (AM1.5 spectrum, 25 °C, normal incidence). Specifically: (i) The incident solar spectrum exhibits pronounced temporal, seasonal, and atmospheric variability—e.g., exhibiting a red−shifted profile at sunrise/sunset and a blue−shifted profile under overcast conditions—which directly modulates the photocurrent ratio between top and bottom sub−cells, thereby inducing current mismatch and substantial degradation in overall conversion efficiency. (ii) The diffuse−to−direct irradiance ratio increases markedly under cloudy conditions or at high latitudes; as diffuse light is predominantly obliquely incident, antireflection coatings and surface texturing—optimized for normal incidence—exhibit elevated reflectivity, further perturbing the effective spectral distribution. (iii) Multiple optical interfaces—including encapsulation glass, adhesive interlayers, and sub−cell junctions—introduce cumulative interference and parasitic absorption effects upon photon propagation. (iv) Sub−cell temperature coefficients often differ substantially (e.g., perovskite top cells and silicon bottom cells exhibit distinct dEg/dT values); consequently, the narrow−bandgap bottom cell experiences accelerated photocurrent decay at elevated temperatures, intensifying current mismatch, while non−uniform thermal distribution may provoke localized hot spots. (v) For bifacial tandem modules, ground albedo contributes significantly to rear−side illumination; however, the spectral composition of reflected light varies with surface albedo characteristics (e.g., snow yields a blue−rich reflection, whereas red brick produces a red−enhanced spectrum), thereby altering the spectral weighting of rear−illuminated bottom−cell photocurrent. (vi) In a single−junction solar cell operating at the maximum power point, the recombination current is typically minimal because nearly all charge carriers are effectively extracted. However, in a tandem solar cell, current mismatch among sub−cells induces substantial recombination, especially in the top cell. If the recombination is radiative, the light re−emitted from the top cell can be absorbed and utilized by the bottom cell, a phenomenon known as luminescent coupling (LC). It is an intrinsic, unavoidable physical process in monolithic tandem structures [101]. Collectively, these factors underscore that optimal light management for tandem cells should target annualized current matching across diverse operational scenarios, not merely peak efficiency under idealized standard test conditions.

In conclusion, optical management in monolithic perovskite/silicon tandems has moved beyond maximizing short−circuit current under standard test conditions. Key advances include textured surfaces (micro−/nano−pyramids) for light trapping, antireflection coatings (e.g., LiF), and optimized TCO layers (e.g., IZO) to reduce parasitic absorption. However, the critical insight is that real−world efficiency depends on annual energy yield, not peak lab performance. Factors such as spectral variation (red−shift at dawn/dusk, blue−shift under clouds), diffuse/direct irradiance ratio, oblique incidence angle, differential temperature coefficients between sub−cells, and ground albedo (for bifacial designs) cause dynamic current mismatch. Importantly, luminescent coupling (LC)—where radiative recombination in the top cell provides additional photons to the bottom cell—partially compensates for mismatch and stabilizes output. Thus, optimal light management must target spectral−robust current matching across diverse outdoor conditions, leveraging simulation and LC effects, rather than a single static design.

## 5. Conclusions and Outlook

In summary, the rapid efficiency advancement of PSTSCs stems from three synergistic pillars: (i) the continuous optimization of perovskite top−cell materials and deposition processes, (ii) the improved passivation and light−trapping capabilities of textured crystalline silicon bottom cells, and (iii) systematic optical management across the entire tandem stack. These combined efforts have pushed monolithic PSTSCs from the laboratory scale to the threshold of industrial viability.

Nevertheless, the path to commercialization is not without competition. Four−terminal perovskite/silicon tandem modules [102] offer distinct advantages: they decouple the two sub−cells electrically, exhibit high tolerance to spectral mismatch, and provide greater flexibility in module design and application scenarios. When comparing actual energy yield rather than simply efficiency under standard test conditions, two−terminal tandems are inherently sensitive to variations in the solar spectrum and incident angle. Under the same nominal efficiency, four−terminal modules often deliver higher actual energy output. However, this picture is nuanced. Experimental and theoretical work by Kohnen et al. demonstrated that the fill factor (FF) of monolithic PSTSCs increases with current mismatch, partially compensating for the loss in short−circuit current density (Jsc) and thereby reducing the sensitivity of power conversion efficiency (PCE) to spectral variations [100]. More recently, the luminescent coupling (LC) effect revealed by Nguyen et al. further strengthens the case for monolithic devices. Over 85% of the photons emitted by the perovskite top cell are reabsorbed by the silicon bottom cell, generating additional photocurrent. This inter−sub−cell energy redistribution not only boosts the overall current output but also mitigates current mismatch caused by fluctuating spectral conditions, enhancing the stable power generation capacity under real−world outdoor and variable environments [43]. Thus, while four−terminal configurations offer operational flexibility, monolithic tandems possess intrinsic photophysical mechanisms that partially offset their spectral sensitivity—a point often overlooked in simplistic comparisons.

Despite these promising advances, several formidable challenges remain on the road to large−scale commercialization.

1. Long−term stability and environmental robustness. The inherent issues of halide phase segregation and ion migration in wide−bandgap perovskites—particularly under combined stressors of humidity, light, heat, and electric field—have not yet been fundamentally resolved. Outdoor field data reveal that early tandem devices with 25% efficiency suffer from rapid degradation under high−temperature and high−light−intensity conditions [103,104]. Addressing this requires the development of novel encapsulation systems that combine chemical inertness with mechanical flexibility, alongside a deeper mechanistic understanding of failure modes under multi−stress coupling (e.g., simultaneous thermal and illumination cycling). Moreover, the stability of self−assembled monolayers (SAMs) and other interfacial layers under operational conditions remains underexplored.
2. Manufacturing compatibility and cost control. Solution−based processing of perovskite top cells still largely relies on spin−coating in inert or cleanroom environments, which is inherently wasteful and difficult to scale. Transitioning to fully printable, high−throughput manufacturing processes (e.g., slot−die coating, blade coating, or vapor−based methods) is essential. In parallel, the replacement of toxic solvents (such as DMF and NMP) with green, low−toxicity alternatives must be prioritized to meet environmental and occupational safety regulations. The compatibility of these processes with industrially textured silicon wafers—which feature micron−scale pyramids—adds another layer of complexity, as achieving conformal, pinhole−free perovskite films on such topographies remains non−trivial.
3. Standardized testing and certification. Current efficiency records are predominantly measured on small−area devices (≈1 cm^2^) under carefully controlled laboratory conditions. For large−area modules (≥100 cm^2^), challenges such as film uniformity, series resistance losses, and current matching across the entire substrate become critical bottlenecks. Furthermore, the International Electrotechnical Commission (IEC) has yet to establish specific certification protocols for perovskite/silicon tandem solar cells, including guidelines for light soaking, thermal cycling, and reverse−bias testing. This lack of standardization hinders the comparability of results across research groups and slows down industrial adoption.
4. Additional hidden challenges. Beyond the issues listed above, the reliable electrical interconnection of large−area tandem modules, the availability of transparent conductive oxides (TCOs) with both high conductivity and low parasitic absorption, and the end−of−life recycling of devices containing lead remain underexplored but practically relevant concerns.

Looking forward, we outline several priority directions for research and development:

Materials innovation: Develop new perovskite formulations with a tunable bandgap in the optimal range (1.65–1.75 eV) and enhanced phase stability. Promising approaches include two−dimensional/three−dimensional (2D/3D) heterostructures to suppress halide segregation, strain engineering to stabilize the perovskite lattice, and high−entropy compositions that exploit configurational entropy for thermodynamic stabilization.

In situ process characterization: Employ advanced in situ characterization techniques—such as synchrotron−based grazing−incidence wide−angle X−ray scattering (GIWAXS), operando optical spectroscopy, and femtosecond transient absorption spectroscopy—to monitor the crystallization kinetics of perovskites on textured silicon surfaces in real time. These insights are essential for rational process optimization and defect reduction.

Flexible and ultra−light tandems: The emergence of flexible and ultra−thin perovskite/silicon tandem cells opens up new application domains, including building−integrated photovoltaics, portable electronics, and aerospace power sources. However, critical challenges must be addressed: the formation of microcracks in silicon wafers thinned to 30 μm under bending stress, the development of robust, flexible transparent electrodes, and the optical management of ultra−thin bottom cells where light absorption is inherently limited. Understanding the light absorption compensation mechanisms in such thin absorbers will be key to maintaining high efficiency under mechanical deformation.

Industrial−scale validation: Collaborative efforts between academia and industry are urgently needed to establish pilot production lines, develop accelerated aging protocols tailored to tandem devices, and generate field performance data under diverse climatic conditions. Only through such validation can laboratory records translate into commercially viable products.

In conclusion, monolithic perovskite/silicon tandem solar cells have reached an exciting juncture where efficiencies above 33% have been demonstrated, yet the transition from record−breaking cells to reliable, cost−effective, and scalable modules demands concerted efforts across materials chemistry, device physics, processing engineering, and standardization. This review has systematically analyzed the key factors enabling the recent efficiency leap, and we hope that the insights and remaining challenges outlined here will guide future research toward the ultimate goal of making perovskite/silicon tandem photovoltaics a cornerstone of the global renewable energy mix.

## Figures and Tables

**Table 1:** Summary of 2T PK/c−Si tandem (PCE > 33%) reported in literature.

Device Structure	PCE (%)	Voc (V)	Jsc (mA/cm^2^)	FF (%)	Ref.
SHJ−based tandem solar cells					
Ag/MgF_2_/IZO/a−Si(p)/a−Si(i)/c−Si/a−Si(i)/nc−Si(n)/ IZO/Me−4PACz/Cs_0.05_FA_0.8_MA_0.15_Pb(I_0.755_Br_0.255_)_3_ + THTZ−H^+^/PDAI/LiF/C_60_/SnO_2_/IZO/Ag/MgF_2_	33.7 ^2^ 34.0 ^1^	1.985	21.02	81.6	[25]
Ag/TCO/nc−Si(p)/a−Si(i)/c−Si/a−Si(i)/nc−SiO_x_(n)/ IZO/MeO−4PACz/FA_0.8_MA_0.15_Cs_0.05_Pb(I_0.76_Br_0.24_)_3_/LiF/EDAI/C_60_/SnO_2_/IZO/Ag/MgF_2_	33.89 ^2^	1.966	20.76	83.0	[22]
Ag/ITO/SiO_2_−NP/SiO_x_/nc−Si(p)/a−Si(i)/c−Si/a−Si(i)/nc−Si(n)/ITO/Me−4PACz/SiO_2_−NP/Cs_0.05_(FA_0.9_MA_0.1_)_0.95_Pb(I_0.8_Br_0.2_)_3_/PCI/C_60_/SnO_x_/IZrO/Ag/LiF	33.28 ^1^	1.993	20.55	81.24	[26]
SHJ Si−cell/ MeO−2PACz/Cs_0.05_(FA_0.77_MA_0.23_)_0.95_Pb(I_0.77_Br_0.23_)_3_ + NIS/MHI/C_60_/SnO_2_/ITO/Ag/MgF_2_	33.2 ^2^	1.996	20.45	81.33	[27]
Ag/ITO/nc−Si(p)/a−Si(i)/c−Si/a−Si(i)/nc−SiO_x_(n)/ IZO/HTL210/Cs_0.02_(FA_0.77_MA_0.23_)_0.98_Pb(I_0.77_Br_0.23_)_3_/LiF/EDAI/C_60_/SnO_2_/IZO/Ag/MgF_2_	34.58 ^2^	1.996	20.80	83.63	[28]
Ag/TCO/nc−Si(p)/a−Si(i)/c−Si/a−Si(i)/nc−SiO_x_(n)/ TCO/RS2/FA_0.8_MA_0.15_Cs_0.05_Pb(I_0.76_Br_0.24_)_3_/LiF/EDAI/C_60_/SnO_2_/IZO/Ag/MgF_2_	34.2 ^2^	1.99	20.7	83.0	[29]
Ag/ITO/a−Si(p)/a−Si(i)/c−Si/a−Si(i)/a−Si (n)/ ITO/A7HPA/Cs_0.05_FA_0.8_MA_0.15_PbI_2.29_Br_0.70_/PI/C_60_/SnO_x_/IZO/Ag/MgF_x_	32.57 ^1,2^ 33.06 ^1^	1.958	19.85	83.77	[30]
Ag/TCO/nc−Si(p)/a−Si(i)/c−Si/a−Si(i)/nc−SiO_x_ (n)/ ITO/SiO_x_/2PACz/Cs_0.05_FA_0.8_MA_0.15_PbI_2.27_Br_0.73_/Pi/C_60_/SnO_x_/IZO/Ag/MgF_x_	33.15 ^1,2^	1.95	20.92	81.07	[31]
Ag/ITO/nc−Si(p)/a−Si(i)/c−Si/a−Si(i)/nc−SiO_x_(n)/ ITO/Me−4PACz/Cs_0.05_FA_0.8_MA_0.15_Pb(I_0.75_Br_0.25_)_3_/2D(PEAI + SbCl_3_)/C_60_/SnO_x_/IZO/Ag/MgF_x_	32.56 ^2^ 33.10 ^1^	1.949	20.51	81.43	[32]
Ag/TCO/nc−Si(p)/a−Si(i)/c−Si/a−Si(i)/nc−SiO_x_ (n)/ ITO/DMMP/Cs_0.05_FA_0.8_MA_0.15_Pb(I_0.75_Br_0.25_)_3_/PI/C_60_/SnO_x_/IZO/Ag/MgF_x_	33.59 ^1,2^ 33.04 ^2^	1.976	20.41	83.26	[33]
Ag/ITO/a−Si(p)/a−Si(i)/c−Si/a−Si(i)/nc−Si(n)/ ITO/Me−4PACz/Cs_0.05_FA_0.8_MA_0.15_Pb(I_0.755_Br_0.255_)_3_/PDAI/LiF/C_60_/SnO_2_/ITO + IZO/Ag/MgF_x_	33.1 ^1^	2.011	20.6	79.7	[34]
Ag/ITO/a−Si(p)/a−Si(i)/c−Si/a−Si(i)/nc−Si(n)/ IZO/CL−SAMs/CsFAMAPb(IBr)_3_/LiF/C_60_/SnO_2_/IZO/Ag/MgF_2_	33.61 ^2^ 34.1 ^1^	1.98	21.0	81.9	[35]
Ag/ITO/a−Si(p)/a−Si(i)/c−Si/a−Si(i)/a−Si(n)/ ITO/CbzNaph/Rb_0.05_Cs_0.1_FA_0.85_Pb(I_0.75_Br_0.25_)_3_ + MLAI/EDAI_2_ + PEAI/C_60_/SnO_x_/IZO/Ag/MgF_x_	32.85 ^2^ 33.22 ^1,2^	2.006	20.23	81.98	[36]
Ag/MgF_x_/IMO/nc−Si(p)/a−Si(i)/c−Si/a−Si(i)/nc−SiO_x_(n)/ nc−Si(n^+^/p^+^)/MeO−2PACz/Cs_0.2_FA_0.8_Pb(I_0.83_Br_0.17_)_3_/PI/C_60_/SnO_2_/IZO/Ag/LiF_x_	32.94 ^1,2^ 33.38 ^1^	1.977	20.95	79.26	[37]
Ag/ITO/nc−Si(p)/a−Si(i)/c−Si/a−Si(i)/nc−SiO_x_(n)/ ITO/Me−4PACz/Cs_0.05_FA_0.8_MA_0.15_Pb(I_0.75_Br_0.25_)_3_ + KSCN/MgF_x_/C_60_/SnO_x_/IZO/Ag/MgF_x_	32.52 ^1,2^ 33.08 ^1^	1.955	21.05	79.01	[38]
TOPCon−based tandem solar cells					
Ag/SiN_x_/Al2O3/poly Si(p)/SiOx/c Si(n)/SiOx/poly Si(n)/ ITO/NiOx/ICL−3Me/Cs_0.05_FA_0.8_MA_0.15_Pb(I_0.75_Br_0.25_)_3_ C_60_/SnO_x_/IZO/Ag/MgF_2_	32.32 ^2^ 33.12 ^1^	2.015	20.22	79.33	[39]
Flexible tandem solar cells					
Ag/MgF_x_/ITO/nc−Si(p)/a−Si(i)/c−Si/a−Si(i)/nc−SiO_x_(n)/ ICO/Me−4PACz/Cs_0.05_FA_0.8_MA_0.15_Pb(I_0.75_Br_0.25_)_3_/Pipl/C_60_/SnO_2_/IZO/Ag/MgF_x_(Flexible cell)	33.60 ^2^	2.015	20.36	81.91	[40]
Ag/TCO/nc−Si(p)/a−Si(i)/c−Si/a−Si(i)/nc−SiO_x_(n)/ ITO/HTL210/FA_0.8_MA_0.15_Cs_0.05_Pb(I_0.76_Br_0.24_)_3_/LiF/EDAI/C_60_/SnO_2_/IZO/Ag/MgF_2_ (Flexible cell)	33.35 ^2^	1.996	19.77	84.5	[41]

^1^ Reverse Scan Efficiency ^2^ Certified Efficiency.

## Data Availability

No new data were created or analyzed in this study.

## Abbreviations

The following abbreviations are used in this manuscript:

SQ	Shockley–Queisser
PCE	Power conversion efficiency
PSTSCs	Perovskite/silicon tandem solar cells
Voc	Open−circuit voltages
FF	Fill factors
Jsc	Short−circuit current
